# Impact of the health education and preventive equipment package (HEPEP) on prevention of *Strongyloides stercoralis* infection among rural communities in Northeast Thailand: a cluster randomized controlled trial

**DOI:** 10.1186/s12889-018-6081-6

**Published:** 2018-10-19

**Authors:** Pokkamol Laoraksawong, Oranuch Sanpool, Rutchanee Rodpai, Tongjit Thanchomnang, Wanida Kanarkard, Wanchai Maleewong, Ratthaphol Kraiklang, Pewpan M Intapan

**Affiliations:** 1 Department of Public Health Administration, Health Promotion, Nutrition, Faculty of Public Health, Khon Kaen University, Khon Kaen, Thailand; 2 Department of Parasitology, Faculty of Medicine, Khon Kaen University, Khon Kaen, Thailand; 3 Research and Diagnostic Center for Emerging Infectious Diseases, Khon Kaen University, Khon Kaen, Thailand; 4 Faculty of Medicine, Mahasarakham University, Mahasarakham, Thailand; 5 Department of Computer Engineering, Faculty of Engineering, Khon Kaen University, Khon Kaen, Thailand

**Keywords:** *Strongyloides stercoralis*, Health education and preventive equipment package, Thailand

## Abstract

**Background:**

Strongyloidiasis is prevalent in northeast Thailand. This study aimed to evaluate the impact of the Health Education and Preventive Equipment Package (HEPEP), a package we developed to improve awareness and aid in the prevention of *Strongyloides stercoralis* infection among rural communities in northeast Thailand.

**Methods:**

This was an intervention trial conducted in 12 villages (six interventions and six controls) in rural areas of northeast Thailand from March 2016 to September 2017. Single stool sample was collected from each participant and examined using agar plate culture (APC) technique. Each participant was interviewed using a pre-tested questionnaire, treated with single dose of ivermectin (200 μg/Kg), and allocated to either the intervention or control group. Members of the intervention group were given “Practices to Prevent Strongyloidiasis” poster and vinyl boards containing information aimed at raising awareness of *S. stercoralis* and strongyloidiasis. In addition, they were given a poster lecture regarding the lifecycle of *S. stercoralis* before being treated with ivermectin. Aside from that, they were also given a protective equipment package. Monthly refresher courses were provided by village health volunteers (VHVs) regarding the health information they had received and proper equipment usage. The control group, on the other hand, was only provided with a five-minute lecture regarding strongyloidiasis. Assessment of new infection was conducted 3 months later in 327 and 318 participants in the intervention group and control group, respectively.

**Results:**

The HEPEP had 41% greater efficacy in preventing *S. stercoralis* infection in the intervention group than the measures taken in the control group (adjusted Odds Ratio (aOR) = 0.59; 95%CI: 0.41 to 0.85, *P-value* = 0.005). The intervention group also scored significantly higher on all aspects of a test of *S. stercoralis* knowledge compared with the control group (mean difference (mean dif.) = 2.89, *P-value* = < 0.05).

**Conclusions:**

The HEPEP was the first model that has been found to be effective in controlling of *S. stercoralis* in rural communities in the northeast Thailand. The results should encourage policy makers and public health personnel to improve control programs, as well as health promotion, with regard to parasites.

**Trial registration:**

Thai Clinical Trials Registry (TCTR), Medical Research Foundation of Thailand, Medical Research Network of the Consortium of Thai Medical Schools: MedResNet (Thailand) (identification number: TCTR20180404002) Registered 4 April 2018 (retrospectively registered).

**Electronic supplementary material:**

The online version of this article (10.1186/s12889-018-6081-6) contains supplementary material, which is available to authorized users.

## Background

Human strongyloidiasis, which is caused by infection with a parasitic nematode of the genus *Strongyloides*, is an important public health problem, especially in tropical and sub-tropical countries [1, 2]. Currently, more than 100 million people are infected with *Strongyloides stercoralis* worldwide [3–5]. *Strongyloides stercoralis* has a complex life cycle, which includes free-living and parasitic cycles, as well as autoinfection [3, 5, 6]. The free-living life cycle enables the parasite to persist in the surrounding environment [7]. In addition, the possibility of autoinfection, together with asymptomatic chronic infection, enables the parasite to persist in humans [7]. Moreover, autoinfection can lead to hyperinfection and disseminated strongyloidiasis [5, 7–9].

Thailand is a tropical country that has an environment suitable for *S. stercoralis* in its free-living phase. This leads to a high risk of human infection during its parasitic phrase. In the northeastern region, the prevalence of *S. stercoralis* infection has been shown to range from 2.5 to 33.3% based on community surveys [2, 10–15]. For example, an eleven-year retrospective hospital-based study showed that the prevalence of infection ranged from 11.0 to 24.3% in the northeast region [2]. Accordingly, strongyloidiasis is considered to be a helminthiasis of public health importance in Thailand requiring the development and implementation of an integrated approach to prevention and control that includes screening, mass treatment, and health education [2]. It has been recommended that these strategies should incorporate multiple interventions to maximize the sustainability of control programs [16]. This paper aimed to evaluate the impact of a Health Education and Preventive Equipment Package (HEPEP) on the prevention of *S. stercoralis* infection among rural communities in northeast Thailand.

## Methods

### Study design

This study was an open-label controlled trial [Thai Clinical Trials Registry (TCTR), Medical Research Foundation of Thailand, Medical Research Network of the Consortium of Thai Medical Schools: MedResNet (Thailand) (identification number: TCTR20180404002)] that aimed to evaluate the impact of the Health Education and Preventive Equipment Package on prevention and control of *S. stercoralis* infection among communities in northeast Thailand from March 2016 to September 2017. Participants from one area served as an experimental group, while those from another area near the first served as the control group.

### Study area and study population

**Fig. 1:**
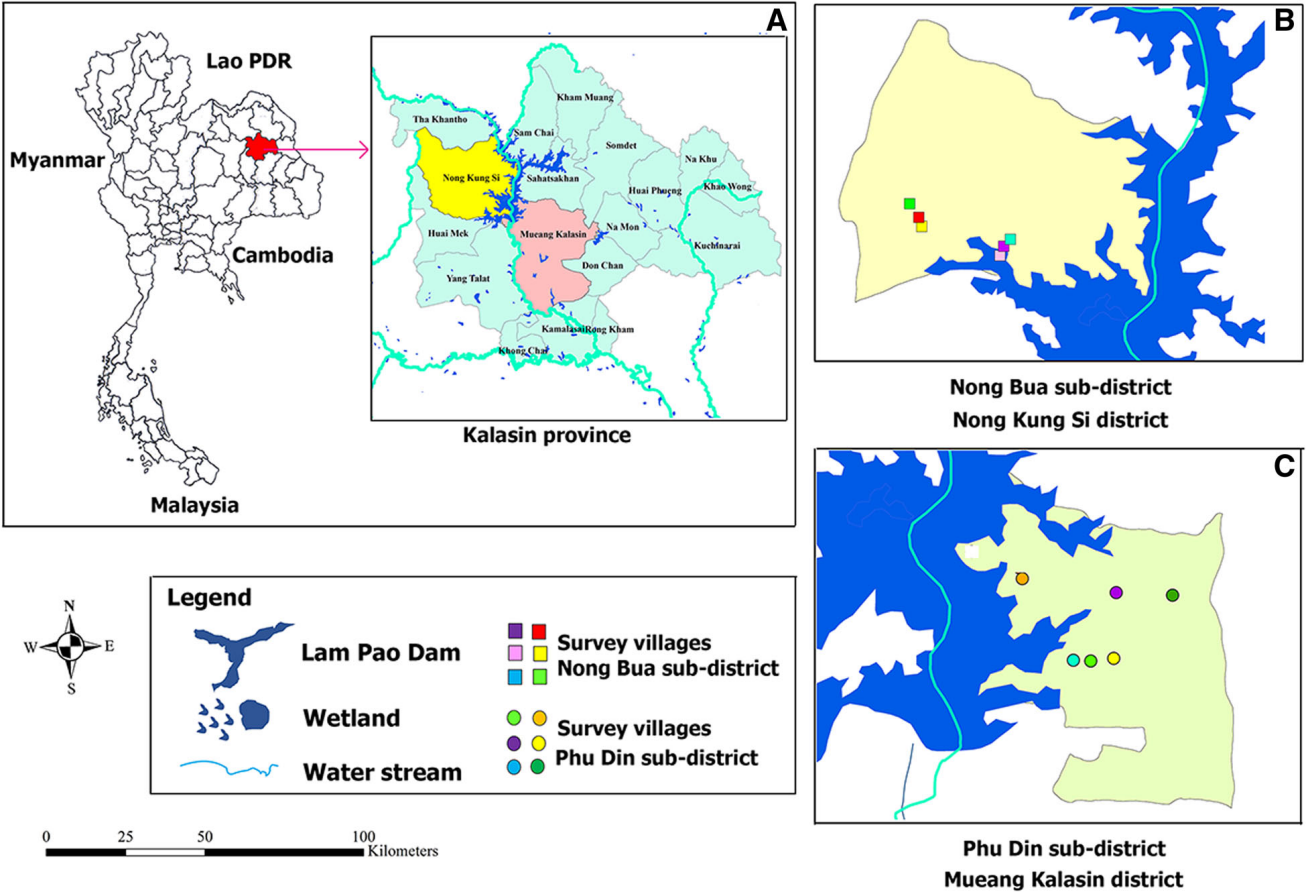
(**a)** geographic map showing Kalasin Province in northeast Thailand and the location of the selected villages in Nong bua (**b**) and Phu Din (**c**) sub-districts

**Fig. 2:**
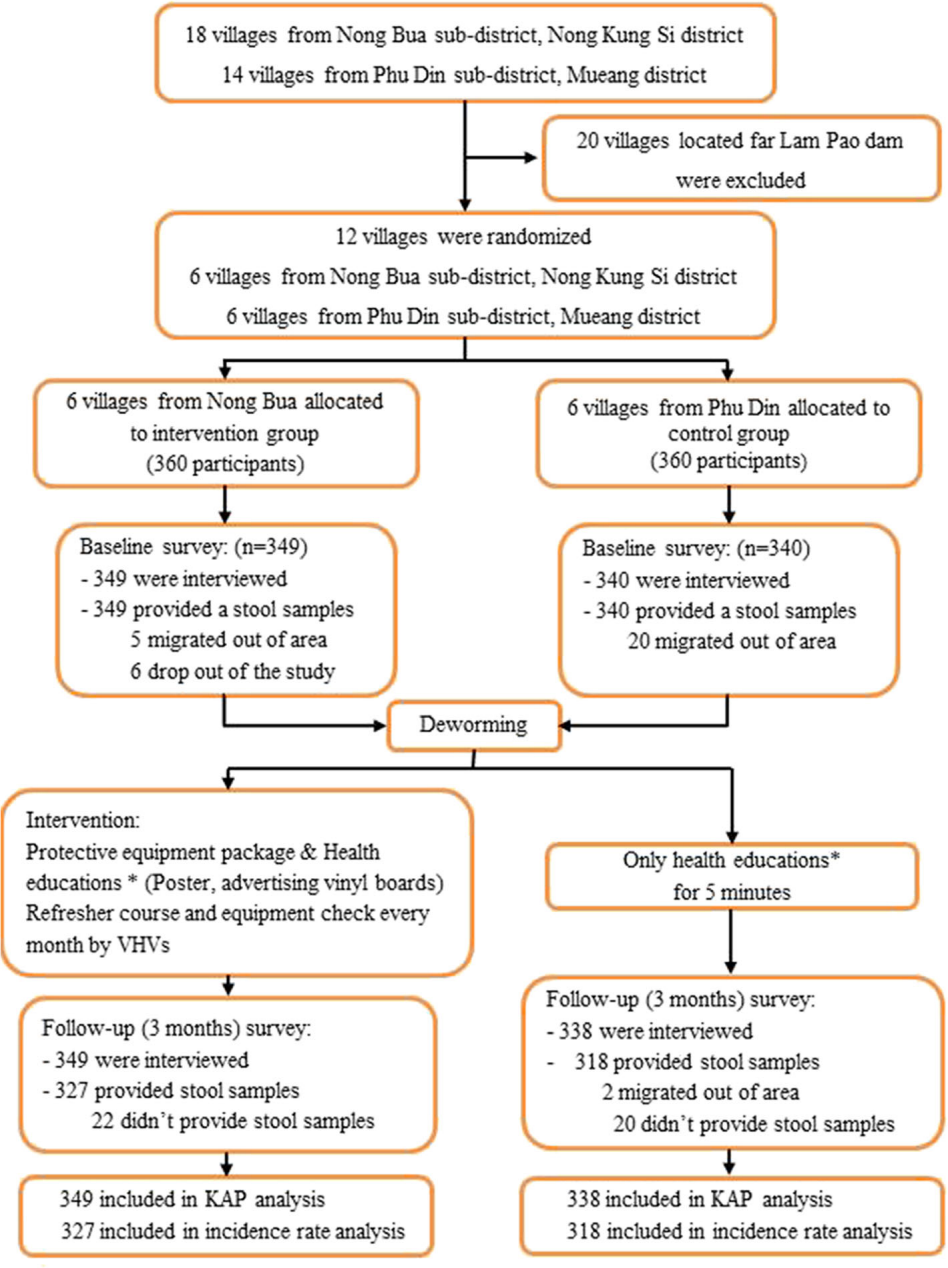
Flow chart of the study’s activities and follow up. *Health education program different between intervention group and control group. KAP: A Knowledge, Attitude and Practices; VHVs: Village health volunteers

This study was carried out in two areas of Kalasin province in northeast Thailand: (1) Nong Bua sub-district in Nong Kung Si district (intervention group) and (2) Phu Din sub-district in Mueang Kalasin district (control group). Both areas are located near Lam Pao dam. Nong Bua sub-district is located at 16.716733^°^ latitude and 103.383900^°^ longitude and Phu Din sub-district is located at 16.643328^°^ latitude and 103.517948^°^ longitude (Fig. 1). Residents of both areas are primarily agriculturists (i.e. working in rice fields, cassava, sugarcane, and Para rubber farms) [17]. The two areas were selected based on data from previous studies showing that the province had a high prevalence of strongyloidiasis [10, 18]. The sample size was determined using the command “clustersampsi, binomial sample size” in STATA Version 10.1 (College Station, Texas: StataCorp LLC). The STATA command that was used was “clustersampsi, binomial samplesize p1(.23) p2(.10) m(30) rho(0.034) alpha(0.05) beta(0.80)”. It was calculated based on the prevalence rate (p_1_) of 23.0% found in a previous study [10], a prevalence rate after added intervention (p_2_) of 10.0% with a 95% confidence interval (*Z* ^2^_∝/2_ = 1.96), 80% confidence interval (Z_β_ = 0.84), design effect of 2, 10 clusters per arm, and an intra cluster correlation (ICC) of 0.034. The calculated sample size was 300 per area. We assumed that the final sample size would be reduced by around 20% due to unavailability of stool on the day of collection, making the adjusted sample size 360 per area. A simple random sampling method was used to select subjects from each sub-district. Subject inclusion criteria were that participants were 1) residents of Nong Bua or Phu Din sub-district and 2) age ≥ 20 years old. Subjects were excluded if they 1) had recently migrating from other areas or 2) dropped out of the study. Subsequently, they were given plastic containers for stool collection with instructions. In the end, a total of 689 subjects returned stool specimens, 349 from the six villages in the intervention group and 340 from the six villages in the control group (Fig. 2).

### Baseline data collection and empirical methods

Baseline data collection included screening for eligibility and *S. stercoralis* infection diagnosis, as well as data from a questionnaire assessing participants’ knowledge and behavior with regard to *S. stercoralis* infection (see Additional file 1). The collection of data regarding demographic, socioeconomic, and environmental factors was conducted between January and May 2017. All participants who were infected with *S. stercoralis* underwent treatment and a subsequent follow-up 21–28 days post treatment (in June 2017). An intervention study was then initiated to evaluate the efficacy of the Health Education and Preventive Equipment Package (HEPEP) on September 2017 (Fig. 2).

### Questionnaire

After giving written consent, research participants were interviewed in person at their homes using a three-part questionnaire which is developed for the present study. The first part consisted of demographic, socioeconomic, and environmental data; habits; and health status. The second part revolved around knowledge regarding *S. stercoralis* consisting of 15 questions. The questions in the second part were designed to test respondents’ knowledge regarding *S. stercoralis* (biology, transmission, symptoms, prevention, and control). The knowledge score was interpreted based on the method employed by Bloom et al. [19]: 13–15 (> 80.00%) = high level of knowledge, 10–12 (60.01–79.99%) = moderate level of knowledge, and 0–9 (0.00 to 60.00%) = low level of knowledge. According, the third part revolved around risk behaviors to *S. stercoralis* infection.

### Stool examination

Stool samples were collected at baseline, follow-up (21–28 days post treatment), and 3 months later in both the control and intervention groups. Clean plastic containers labeled with the participants’ names and code numbers were distributed to all participants by VHVs in each village. On the following day, the full containers were returned to the field staff who performed agar plate culture (APC), as described by Koga et al. [20], for detection of *S. stercoralis* infection. Two to 3 grams of stool was added to 1% nutrient agar in a plate that was 9 cm in diameter. The agar plate was incubated at 25-27^๐^C for 3–5 days in a dark box and was examined under a stereomicroscope on either the third or fifth day [20]. The plates were transported to the Parasitology laboratory at the Khon Kaen University Faculty of Medicine and observed under a stereomicroscope by qualified parasitologists. A negative result was recorded when *S. stercoralis* was not detected within 5 days of incubation.

### Deworming

At baseline, follow-up, and three-months, participants who were infected with *S. stercoralis* were listed accordingly and received ivermectin (200 μg/Kg body weight, single dose; Atlantic Laboratories Corporation Ltd., Samut Prakan, Thailand). All participants were administered the medication under observation by a researcher and medical officer. There were no complaints from the participants during ivermectin administration.

### Preventive equipment package and follow up

Health education was provided to participants in both groups, but the intervention group was provided with a preventive equipment package (gloves and boots) and detailed information regarding *S. stercoralis* in the form of a “Practices to Prevent Strongyloidiasis” poster (size 29 × 40 cm) to be hung in participants’ houses (see Additional file 2), vinyl boards containing information on *S. stercoralis* and strongyloidiasis (size 2 × 3 m) to be posted in each village (see Additional file 3), and a “*S. stercoralis* Life Cycle” poster (size 90 × 120 cm) (see Additional file 4) with a 20-min lecture explaining its contents. Subsequently, the intervention group was given a refresher course and their use of the equipment that was provided was checked every month by village health volunteers (VHVs). The control group was provided with only a five-minute lecture regarding human *S. stercoralis* infection. The participants from both areas were given follow-up *S. stercoralis* examinations and interviewed over the following 3 months (Fig. 2). The HEPEP-related follow-ups consisted of monthly visits to the villages in question by researchers and VHVs to observe the participants’ practices with regard to wearing shoes and gloves while working on their farms (see Additional file 5). In addition, the VHVs reminded the participants about *S. stercoralis* transmission monthly via a broadcast tower in each village (Fig. 2).

### Statistical analysis

Frequency, percentage, mean, and standard deviation (SD) were used to describe demographic characteristics. Prevalence of *S. stercoralis* infection was described in terms of proportion and 95% confidence interval (95% CI). To investigate the impact of Health Education and Protective Equipment Package on *S. stercoralis* infection, the prevalence of *S. stercoralis* infection in the intervention group and that in the control group were compared using logistic regression and a generalized estimating equation (GEE). To investigate the impact of the Health Education and Protective Equipment Package on knowledge scores*,* knowledge scores at baseline and 3 months were compared using pair *t*-test. To investigate the impact of the Health Education and Protective Equipment Package on behavior in each group, behavior at baseline and 3 months were compared using a paired McNemar’s test. For adjusting possible confounders, all variables with a *P-*value less than 0.1 in the univariate analysis were selected. Additionally, the knowledge scores in the intervention and the control group were compared using a *t*-test. A *P-value* of less than 0.05 was considered statistically significant. The statistical analysis was conducted using the STATA package version 10.1 (College Station, Texas: StataCorp LLC).

## Results

### Demographic characteristics

A total of 689 study participants, 349 from Nong Bua sub-district (intervention group) and 340 from Phu Din sub-district (control group), were enrolled in the study. Three hundred twenty-three (46.88%) of the participants were male and 366 (53.12%) were female. The mean age (±SD) was 51.19 (±12.04) years (range = 20–87 years). Four hundred sixty-seven of the participants (67.77%) had graduated from primary school and 472 (68.51%) were agriculturists. Most of the participants and household income lower than $250 per month (according to the exchange rate as of 1 Nov 2017; 564 participants; 81.86%) ($250 per month is the poverty line in Thailand) [21]. The average household income (±SD) was $167.54 (±214.15) (range = 0–1757.58$). Most of the participants (495; 71.84%) were healthy. Larva currens were observed on the skin of 14 participants (2.03%). With regard to participants’ residential environments, 441 (64.01%) reported damp soil around their houses and 494 (71.70%) had one or more pets. Most of the participants (688; 99.85%) used a cesspool and septic tank cleaner for feces management (Table 1).

**Table 1:** Baseline characteristic of participants in the intervention (HEPEP) and control group

Variables	Intervention (*n* = 349)	Control (*n* = 340)	Total (*n* = 689)	*P*-value for tests of between-group difference
	Number (%)	Number (%)	Number (%)	
Individual characteristic				
Gender				0.058
Male	176 (50.43)	147 (43.24)	323 (46.88)	
Female	173 (49.57)	193 (56.76)	366 (53.12)	
Age				< 0.001
Mean ± SD (Min:Max)	49.40 ± 11.81 (20:78)	53.03 ± 12.01 (20:87)	51.19 ± 12.04 (20:87)	
Education levels				0.177
Graduated or higher	14 (4.01)	17 (5.00)	31 (4.50)	
Diploma	7 (2.01)	8 (2.35)	15 (2.18)	
Grade 10–12	56 (16.04)	37 (10.88)	93 (13.50)	
Grade 7–9	36 (10.32)	32 (9.41)	68 (9.87)	
Primary school	232 (66.47)	235 (69.12)	467 (67.77)	
No formal education	4 (1.15)	11 (3.24)	15 (2.18)	
Occupations				< 0.001
Trade/ business owner	28 (8.02)	103 (30.29)	131 (19.01)	
Government/private officer	13 (3.72)	21 (6.18)	34 (4.93)	
Student	1 (0.29)	1 (0.29)	2 (0.29)	
Agriculturalist	298 (85.39)	174 (51.18)	472 (68.51)	
Other (Elderly/Housewife)	9 (2.58)	41 (12.06)	50 (7.26)	
BMI				0.089
< 18.50	19 (5.44)	31 (9.12)	50 (7.26)	
18.50 to 24.99	199 (57.02)	171 (50.29)	370 (53.70)	
25.00 to 29.99	108 (30.95)	121 (35.59)	229 (33.24)	
≥ 30.00	23 (6.59)	17 (5.00)	40 (5.80)	
Mean ± SD (Min: Max)	24.10 ± 3.81 (15.06: 36.72)	23.94 ± 4.07 (13.12: 44.82)	24.02 ± 3.94 (13.12: 44.82)	0.608
Household income ($)				0.393
< 250 $	290 (83.10)	274 (80.59)	564 (81.86)	
≥ 250$	59 (16.90)	66 (19.41)	125 (18.14)	
Mean ± SD (Min:Max)	160.42 ± 199.15 (0: 1696.97)	174.86 ± 228.58 (0: 1757.58)	167.54 ± 214.15 (0: 1757.58)	0.377
Marital status				0.71
Single	14 (4.01)	16 (4.71)	30 (4.35)	
Married	312 (89.40)	306 (90.00)	618 (89.70)	
Devoted	23 (6.59)	18 (5.29)	41 (5.95)	
Underlying diseases				0.006
No	267 (76.50)	228 (67.06)	495 (71.84)	
Yes	82 (23.50)	112 (32.94)	194 (28.16)	
Larvae currens				0.961
No	342 (97.99)	333 (97.94)	675 (97.97)	
Yes	7 (2.01)	7 (2.06)	14 (2.03)	
Residential environment				
Has damp soil around house area				0.372
No	120 (34.38)	128 (37.65)	248 (35.99)	
Yes	229 (65.62)	212 (62.35)	441 (64.01)	
Flooding in area				0.834
No	341 (97.71)	333 (97.94)	674 (97.82)	
Yes	8 (2.29)	7 (2.06)	15 (2.18)	
Presence of pet in house				< 0.001
No	125 (35.82)	70 (20.59)	195 (28.30)	
Yes	224 (64.18)	270 (79.41)	494 (71.70)	
Type of toilet				0.311
Cesspool	349 (100.00)	339 (99.71)	688 (99.85)	
Pit latrines	0 (0.00)	1 (0.29)	1 (0.15)	
Feces management				0.311
Septic tank cleaner	349 (100.00)	339 (99.71)	688 (99.85)	
Fertilizer	0 (0.00)	1 (0.29)	1 (0.15)	
Knowledge scores^a^				
Poor (0.00 to 60.00)	59 (16.91)	85 (25.00)	144 (20.90)	0.007
Moderate (60.01 to 79.99)	132 (37.82)	135 (39.71)	267 (38.75)	
Good (80.00 to 100.00)	158 (45.27)	120 (35.29)	278 (40.35)	
Mean ± SD (min:max)	73.81 ± 11.11 (40.00:100.00)	69.51 ± 17.03 (0:93.33)^a^	71.69 ± 14.48 (0.00:100.00)^a^	< 0.001
Behaviors				
Direct contact with soil				0.099
No	17 (4.87)	28 (8.24)	45 (6.53)	
Yes	332 (95.13)	312 (91.76)	644 (93.46)	
Area in which bare feet come in contact with soil^b^	*n* = 332	*n* = 312	*n* = 644	< 0.001
Own Residence	63 (18.97)	122 (39.10)	185 (28.73)	
Own Farm	255 (76.81)	168 (53.85)	423 (65.68)	
Others’ farms	14 (4.22)	22 (7.05)	36 (5.59)	
Use of animal fertilizer				0.007
No	61 (17.48)	88 (25.88)	149 (21.63)	
Yes	288 (82.52)	252 (74.12)	540 (78.37)	
Steroid use				0.014
No	284 (81.38)	250 (73.53)	534 (77.50)	
Yes	65 (18.62)	90 (26.47)	155 (22.50)	
Defecation into surrounding environment				< 0.001
No	57 (16.33)	124 (36.47)	181 (26.27)	
Yes	292 (83.67)	216 (63.53)	508 (73.73)	

^a^Number of participants in the control group = 338 at 3-month assessment and number of total participants = 687

^b^Number of participants followed by the participants who came into direct contact with soil

Two hundred seventy-eight of 689 the participants (40.35%) had adequate knowledge regarding *S. stercoralis* infection. The overall average knowledge score at baseline assessment was 71.69 ± (14.48; range = 0–100), with an average score of 73.81 (±11.11; range = 40–100) in the intervention group and 69.51 (±17.03; range = 0–93.33) in the control group (Table 1). In terms of participant behavior, 644 (93.46%) had direct contact with soil, 423 (65.68%) of whom had contact with soil in the area in which they farmed. Additionally, 540 participants (78.37%) used animal dung as fertilizer. One hundred fifty-five (22.50%) had used steroid drugs in the past. Importantly, 508 participants (73.73%) reported that they sometimes defecated into the surrounding environment, rather than using a latrine (Table 1). Differences in age, occupation, underlying diseases, presence of a pet in the house, area in which there was direct contact with soil, and use of animal dung fertilizers were statistically significant between participants in the intervention area and the control area (Table 1).

### Prevalence of *S. stercoralis* infection at baseline

**Fig. 3:**
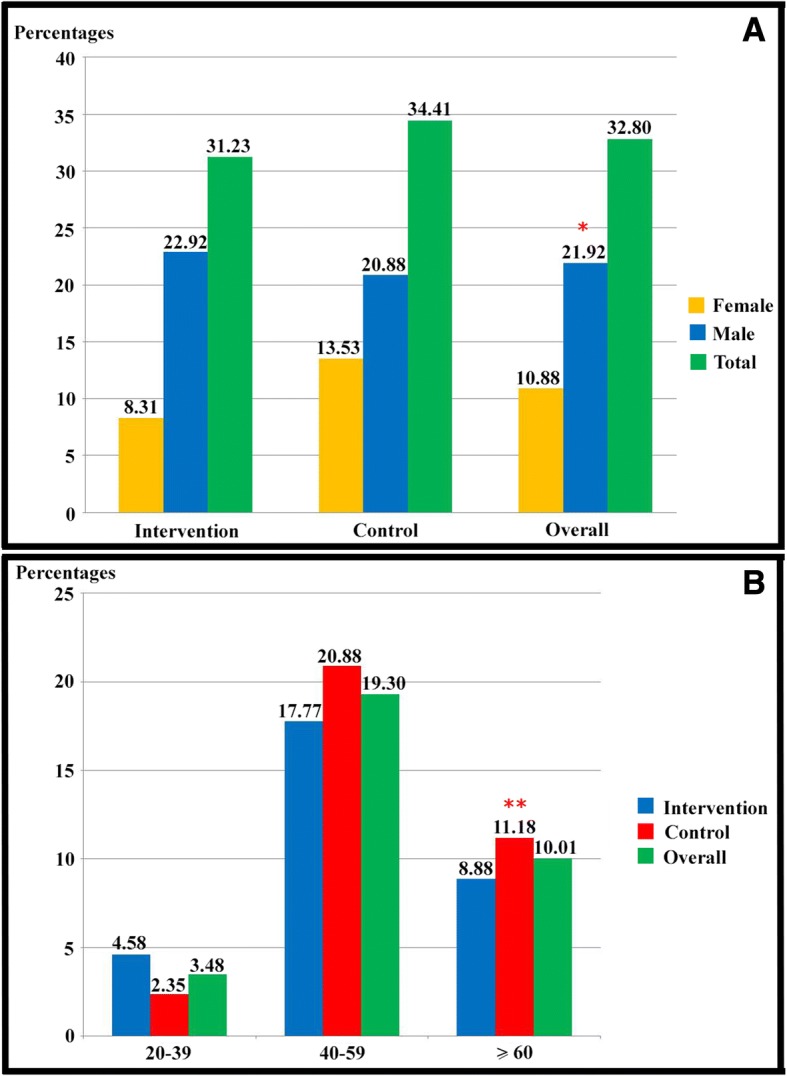
Prevalence of *S. stercoralis* infection at the baseline assessment (**a**) prevalence of *S. stercoralis* infection at the baseline assessment classified by sex, (**b**) prevalence of *S. stercoralis* infection at the baseline assessment classified by age groups ^*^ Statistically significant difference, *P-value* <  0.001 ^**^ Statistically significant difference, *P-value* <  0.05

Two hundred twenty-six (32.80%; 95%CI: 29.29 to 36.32) of the participants were found to be positive for *S. stercoralis* infection according to APC (Fig. 3). The positive rate was higher in male (21.92%) than in female (10.88%) participants. The peak infection rate was found in 40–59 year-old participants (19.30%) (Fig. 3). The baseline prevalence of *S. stercoralis* infections in the intervention group and the control group were comparable 31.23% (95%CI: 26.40 to 36.38) and 34.41% (95%CI: 29.37 to 39.73), respectively with no statistically significant difference (Fig. 3). Twenty-eight days after treatment (follow-up), individual fecal samples of all participants were examined using APC. The prevalence of *S. stercoralis* infection had been reduced to 0% in both groups.

### Impact of the health education and preventive equipment package on the prevalence of *S. stercoralis* at a three-month assessment

**Fig. 4:**
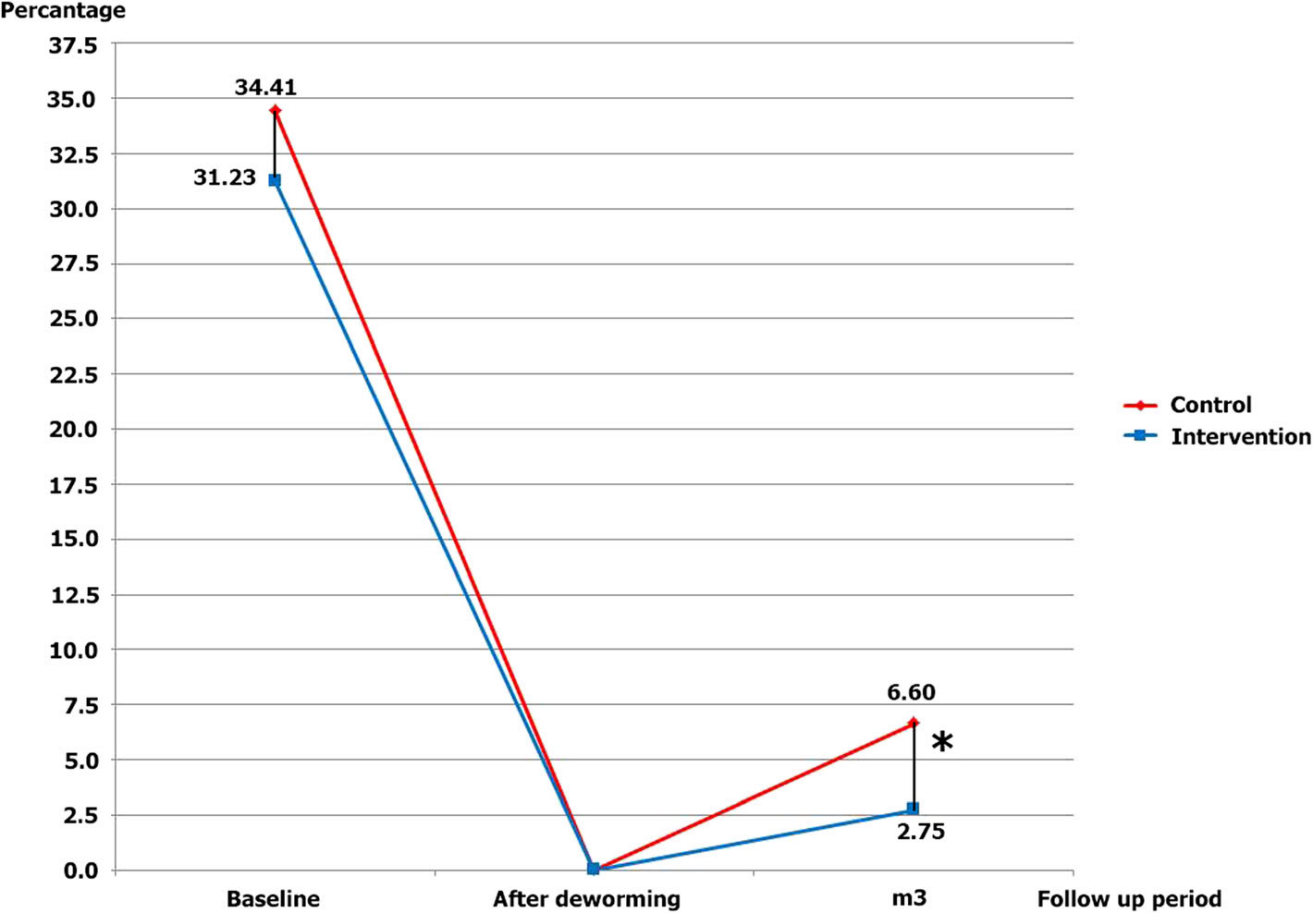
Prevalence and incidence of *S. stercoralis* infection among the intervention and control groups. ^*^Statistically significant difference, *P-value* < 0.05

Three months after treatment, all participants were examined for the presence of *S. stercoralis* infection using APC. The prevalence of *S. stercoralis* infection in the intervention group and that in the control group were 2.75% (9/327) (95%CI: 1.27 to 5.16) and 6.60% (21/318) (95%CI: 4.13 to 9.92), respectively (Fig. 4). There was a statistically significant difference in the prevalence of *S. stercoralis* infection between the intervention group and control group. The efficacy of the HEPEP in the prevention of *S. stercoralis* infection was 60% according to univariable analysis (cOR 0.40; 95%CI: 0.18 to 0.89, *P-value* = 0.02) and 41% according to multivariable analysis (aOR 0.59; 95%CI: 0.41 to 0.85, *P-value* = 0.005; Fig. 4, Table 2).

**Table 2:** Effect of the health education and protective equipment package on prevalence of *S. stercoralis* infection at three-month assessment using a generalized estimating equation (GEE)

Outcome variable	Unadjusted	Adjusted
	cOR (95% CI)	aOR^a^ (95% CI)
*S. stercoralis* prevalence	0.40 (0.18 to 0.89)^**^	0.59 (0.41 to 0.85)^**^

Remark: ^a^Odds ratios adjusted for sex, age, education level, occupation, household income ($), underlying diseases, history of larva currens, presence of a pet in the house, direct contact with soil, use of animal fertilizer, and baseline *S. stercoralis* prevalence

**Statistically significant difference, *P-value* < 0.05

### Impact of the health education and preventive equipment package on the knowledge of the participants in both groups

The knowledge of the participants from both groups was assessed at baseline and 3 months after starting the intervention study. The average knowledge scores in the intervention was statistically significant higher at 3 months than at baseline (83.82 [±10.35] vs 73.81 [±11.11]; mean difference [mean dif.] = 10.01, 95%CI: 8.58 to 11.44, *P-value* = < 0.001; Table 3). Participants in the control group also had a significantly higher knowledge score at 3 months than at baseline (76.63 [±13.02] vs 69.51 [±17.03]; mean dif. = 7.12, 95%CI: 5.12 to 9.12 *P-value* = < 0.001; Table 3). In terms of comparison between the two groups, the intervention group had a significantly higher increase in average knowledge score (10.01 [±0.73]) than the control group (7.12 [±1.02]) at 3 months (mean dif. = 2.89, 95%CI: 0.45 to 5.33, *P-value* = 0.021; Table 3).

**Table 3:** *Strongyloides stercoralis* knowledge scores at baseline and follow-up (three-month assessment post-deworming)

Variables	Intervention (*n* = 349)	Control (*n* = 338)	Mean difference between group difference^a^
	Mean (SD)	Mean (SD)	Mean (95%CI)
Baseline assessment			
Knowledge scores	73.81 (11.11)	69.51 (17.03)	4.24 (2.15 to 6.45)*
3 month assessment			
Knowledge scores	83.82 (10.35)	76.63 (13.02)	7.19 (5.43 to 8.95)*
Mean difference between baseline and 3 month difference^b^	10.01 (0.73)	7.12 (1.02)	2.89 (0.44 to 5.33)**

*Statistically significant difference, *P*-value < 0.001

**Statistically significant difference, *P*-value < 0.05

^a^Compared knowledge scores between intervention group and control group using t-test

^b^Compared knowledge scores between baseline and three-month assessment within intervention group and control group using Pair t-test

### Impact of the health education and preventive equipment package on the behavior of participants at three months post-intervention

After starting the intervention, the increased knowledge scores of participants in the intervention group translated into behavioral changes in the areas of direct soil contact, use of animal dung fertilizer, use of steroid drugs, and defecation into the surrounding environment (Table 4). In the intervention group, the participants were significantly less likely to have direct contact with soil (mean dif. = 8.88; 95%CI: 4.70 to 13.07), use animal dung fertilizer (mean dif. = 51.86; 95%CI: 45.76 to 57.96), use steroid drugs (mean dif. = 7.45; 95%CI: 2.26 to 12.63), or defecate into the surrounding environment (mean dif. = 27.51; 95%CI: 21.56 to 33.45) compared with the baseline assessment (Table 4). The increased knowledge scores in the control group translated into behavioral changes in the areas of direct soil contact, use of animal dung fertilizer, use of steroid drugs, and defecation into the surrounding environment (Table 4). At the follow-up, participants in the control group were significantly less likely to have direct contact with soil (mean dif. = 6.21; 95%CI: 1.75 to 10.68), use animal dung fertilizer (mean dif. =37.27; 95%CI: 31.04 to 43.52), use steroid drugs (mean dif. = 8.29; 95%CI: 2.68 to 13.88), or defecate into the surrounding environment (mean dif. =31.06; 95%CI: 24.79 to 37.34) compared with the baseline assessment (Table 4). Changes with regard to participants’ use of steroid drugs and whether or not they defecated into the surrounding environment differed significantly between the intervention and control group at 3 months post-intervention (Table 4).

**Table 4:** Behavior factors at 3 months assessment after starting a full health education program in the intervention group and receiving a lecture in the control group

Behaviors	Intervention			Control			Odds Ratios (95%CI) Compare between intervention and control group at 3 month ^c^
	Baseline (*n* = 349)	3 month (*n* = 349)	Difference between proportions	Baseline (*n* = 338)	3 month (*n* = 338)	Difference between proportions	
	n (%)	n (%)	difference (95%CI)^a^	n (%)	n (%)	difference (95%CI)^b^	
Directly contacted soil							
Yes	332 (95.13)	301 (86.25)	8.88 (4.70 to 13.07)*	310 (91.71)	289 (85.50)	6.21 (1.75 to 10.68)**	0.94 (0.61 to 1.44)
Animal fertilizer use							
Yes	288 (82.52)	107 (30.66)	51.86 (45.76 to 57.96)*	250 (73.96)	124 (36.69)	37.27 (31.04 to 43.52)*	1.31 (0.95 to 1.78)
Steroid drug use							
Yes	65 (18.62)	39 (11.17)	7.45 (2.26 to 12.63)**	88 (26.04)	60 (17.75)	8.29 (2.68 to 13.88)**	1.71 (1.11 to 2.65)**
Defecation into surrounding environment							
Yes	292 (83.67)	196 (56.16)	27.51 (21.56 to 33.45)*	214 (63.31)	109 (32.25)	31.06 (24.79 to 37.34)*	0.37 (0.27 to 0.51)*

^a^Mean difference in intervention group between baseline and three-month assessment after deworming using pair McNemar’s test

^b^Mean difference in control group between baseline and three-month assessment after deworming using pair McNemar’s test

^c^Compared between intervention and control group at 3 months using a chi-squared test

*Statistically significant difference, *P-value* < 0.001

**Statistically significant difference, *P-value* < 0.05

## Discussion

The Health Education and Preventive Equipment Package (HEPEP) in this study demonstrated a 41% efficacy in the interruption *S. stercoralis* infection and transmission in a rural community in northeast Thailand. The reduction in the infection rate was correlated with increased knowledge scores and improvements in personal hygiene practices. Additionally, this is the first effective model of *S. stercoralis* control in adults in a rural community in Thailand. This result was similar to those of other studies that examined control of soil-transmitted helminthes in children [22–24].

At baseline, 32.80% of the participants were found to have *S. stercoralis* infection, which was higher than in previous studies [12–15, 25–28]. Variations in examination techniques, environmental sanitation, socioeconomic factors, and education levels of the participants likely contributed to this difference [29–31]. Participants aged 40–59 years had a 19.30% prevalence of *S. stercoralis* infection, which was higher than in other age groups. Older adults have been shown to be at higher risk for *S. stercoralis* infection due to their having been exposed to contaminated soil over a longer period of time [2, 28].

At the three-month assessment, the prevalence of *S. stercoralis* infection had increased from 0% (after deworming) to 2.75% in the intervention group. In addition, the prevalence of *S. stercoralis* infection in the control group (the participants in which were provided with only a five-minute lecture) had increased from 0% (after deworming) to 6.60%. This study found that the HEPEP was effective in preventing *S. stercoralis* infection.

Although nearly all participants in both groups had flush latrines (cesspool) in their house (99.85%), the prevalence of *S. stercoralis* infection was still high. This suggests that improvement to sanitation infrastructure alone would not be sufficient to reduce the prevalence of *S. stercoralis* infection, as residents do not always use a latrine [32, 33]. Most of the participants were agriculturists and defecated into surrounding environment while working on their farm. As has been previously reported in Vietnam and Lao PDR, the presence of latrines alone is not sufficient to decrease the prevalence of helminthiasis in rural communities if fresh feces are used as fertilizer [34]. Furthermore, a lack of knowledge regarding *S. stercoralis* transmission is an important factor that increases *S. stercoralis* transmission among participants. This study showed that the average knowledge score of participants in the intervention group (received HEPEP) at the three-month assessment was 2.89 points higher than that of participants in the control group. Furthermore, a high knowledge score was associated with a decrease in the prevalence of *S. stercoralis* infection and behavior changes that resulted in decreased infection, which was similar to the results of previous studies [22, 24]. However, the limitation of this study was its short duration (3 months of assessments). Thus, we plan to continue conducting research to assess the long-term effectiveness of the HEPEP (once per year).

## Conclusions

The Health Education and Preventive Equipment Package (HEPEP) was developed and distributed to rural communities in Kalasin province in northeast Thailand as the first health education program aimed at controlling *S. stercoralis* infection in this region. The HEPEP proved effective, especially in terms of preventing *S. stercoralis* infection. The HEPEP may also be a useful model for controlling other soil-transmitted nematode parasites that infect humans via the same route, especially hookworms in endemic areas of southern Thailand.

Despite the implementation of an intensive national parasite control program in rural areas of northeast Thailand decades ago, strongyloidiasis is still highly prevalent and is sympatric with opisthorchiasis. The results of this study support the argument that there is an urgent need to start an integrated and effective *S. stercoralis* control program using the HEPEP supplemented with long-term follow-up.

## Additional files

Additional file 1: Research Questionnaire, a questionnaire assessing participants’ knowledge and behavior. (PDF 358 kb)

Additional file 2: Poster, a “Practices to Prevent Strongyloidiasis” poster. (TIF 1851 kb)

Additional file 3: Vinyl boards, a vinyl boards containing information on *S. stercoralis* and strongyloidiasis. (TIF 761 kb)

Additional file 4: Life Cycle poster, a “*S. stercoralis* Life Cycle” poster. (TIF 2012 kb)

Additional file 5: The HEPEP-related follow-ups, a: equipment package (gloves and boots), b: “practice to prevent strongyloidiasis” poster, c: Lecture activity of human strongyloidiasis prevention by using *S. stercoralis* life cycle poster, d: *S. stercoralis* and strongyloidiasis advertising vinyl boards containing information on *S. stercoralis* and strongyloidiasis to promote in each village, e and f: checked equipment using every month by village health volunteers and researchers. (TIF 5464 kb)

## Abbreviations

95%CI: 95% confidence interval
aOR: Adjusted Odds Ratio
APC: Agar plate culture
BMI: Body mass index
cOR: Crude Odds Ratio
GEE: Generalized estimating equation
HEPEP: The Health Education and Preventive Equipment Package
mean dif.: Mean difference
SD: Standard deviation
VHVs: Village health volunteers
μg/Kg: Microgram per kilogram
