# Supplementary material for: Impact of the health education and preventive equipment package (HEPEP) on prevention of Strongyloides stercoralis infection among rural communities in Northeast Thailand: a cluster randomized controlled trial
Source: BMC Public Health. 2018 Oct 19;18:1184. doi: 10.1186/s12889-018-6081-6 (PMC6194667; doi:10.1186/s12889-018-6081-6)
Supplement: Supplementary file 1 — Research Questionnaire, a questionnaire assessing participants’ knowledge and behavior. (PDF 358 kb) [file 12889_2018_6081_MOESM1_ESM.pdf]

CODE						

The Research Questionnaire

Knowledge Attitude and risk behavior of *S. stercoralis* infection

.... District, Kalasin Province

Part 1: Demographic data

Description: please**,** add the sign (✓) in front of the blank ( ) which you selected

**1. Gender:**	**Gen**
( ) 1. Male ( ) 2. Female	1 = male 2 = Female
**2. Birth date (dd/mm/yyyy)**...	**Birth** (D/M/Y)……………
**Age** : ………………………….…years	**Age** (number) ………….
**3. Weight**................................. Kilogram	**Weight** (number) ………
**4. Height** ....Centimeter	**Height** (number) ………
**5. Education levels**	**Edu**
( ) 1. No formal education	6 = No formal education
( ) 2. Primary school	5 = primary school
( ) 3. Grade 7-9	4 = grade 7-9 school
( ) 4. Grade 10-12	3 = grade 10-12 school
( ) 5. Diploma	2= diploma
( ) 6. Graduated or higher	1 = graduated or higher
**6. Occupations**	**Occ**
( ) 1. Trade/ business owner	1 = trade / owner business.
( ) 2. Government/private officer	2= government/ private office
( ) 3. Student	3 = student
( ) 4. Agriculturalist	4 = Agriculturalist
( ) 5. other (Elderly/Housewife) specific....................	5 = others………………..
**7. Marital status**	**Status**
( ) 1. Single	1= single
( ) 2. Married	2 = married
( ) 3. Devoted	3= devoted

**8. Household income**..Bath	**<u>Inc (number)</u>** ……………
**9. Do you have underlying disease?**	**<u>Undz</u>**
( ) 1. No ( ) 2. Yes specific …………..	0= no 1= yes…………..
**10. Have you ever transplant organs? (i.e. kidney, heart, liver etc.)**	**<u>transor</u>**<br>0= no
( ) 1 No	1=yes
( ) 2. Yes, specific (organ)	**Specific…………………...**
**11. Have you ever cured *S. stercoralis* without stool examination**?	**<u>Druguse</u>**<br>0= not use
( ) 1. No (pass 12) ( ) 2. Yes specific …………..	1= use
**12. How often do you take helminthic drugs (albendazole, mebendazole or ivermectin)?**	**<u>Drugfe</u>**<br>1= every 3 months
( ) 1. Every 3-months	2= every 6 months
( ) 2. Every 6-months	3 = every 12 months
( ) 3. Every 12-months	4 =other …………………
( ) 4. Other specific	
**13. Have you ever had a stool examination for *S. stercoralis* infection?**	**<u>usedetect</u>**<br>0 = not use detect (pas
( ) 1. No (why)…………………. (pass no14, 15)	no.14, 15)
( ) 2. Yes 1 year ago	1 = use 1 year ago
( ) 3. Yes 2-3 years ago	2 = use 2-3 year ago
( ) 4. Yes more 3 years ago	3 = use >3 year ago
**14. If you have ever had a stool examination. Was you found *S. stercoralis*?**	**<u>Result</u>**<br>1= don’t know
( ) 1. Don’t know/ Can’t remember (pass 15)	2 = not found
( ) 2. Not found (pass 15)	3 = found
( ) 3. Found	
**15. You have ever had a *S. stercoralis* infection. Have you been treated?**	**<u>Treat</u>**<br>0 = no …………………
( ) 1. No because..	1 = yes
( ) 2. Yes	

**16. Have you ever been larvae currens on skin?**	**<u>Larvae currens</u>**
( ) 1. No ( ) 2. Yes specific ……………	0 = no 1 = yes ………..
**17. What kind of toilet in your home?**	**<u>Type of toilet</u>**
( ) 1. Cesspool	1= Cesspool
( ) 2. Pit latrines	2= Pit latrines
**18. How do you manage stool in your latrine?**	**<u>Faecesman</u>**
( ) 1. Septic tank cleaner	1= employ host manage
( ) 2. Fertilizer	2= fertilizer
( ) 3. Other specific ……………	3= other ………………
**19. Do you have a pet (dog/cat) in your house?**	**<u>pet</u>**
( ) 1. No (pass no. 20, 21)	0= no (pass no 20, 21)
( ) 2. Dog……………..	1 =dog (………………)
( ) 3. Cat	2 = cat (……………….)
**20. Do you ignore your pets defecated surround house area?**	**<u>petfe</u>**
	0 = no
( ) 1. No ( ) 2. Yes	1 = yes
**21. Have you ever cured helminths in your pets?**	**<u>treatpet</u>**
( ) 1. No ( ) 2. Yes	0 = no 1 = yes
Frequency………………………………………	Freq………………………
The last date……………………………………	Date………………………
**22. Do you have a damp soil around house area?**	**<u>Area</u>**
( ) 1. No	0 = no
( ) 2. Yes specific area……………………	1 = yes …………………
**23. Have you been flooding in your house?**	**<u>Flooding</u>**
( ) 1. No	0 = no
( ) 2. Yes specific	1 = yes
Frequency..................................../years	Freq………………………
Duration of flooding.......................................	Time………………………

Part 2: The knowledge and Attitude of *S. stercoralis* and strongyloidiasis

2.1 The knowledge of *S. stercoralis* and strongyloidiasis

**Topic**	**Yes**	**No**
1. *S. stercoralis* is a tapeworm**.**		
2. The larvae of *S. stercoralis* can live in soil and directly penetrate to human skin.		
3. *S. stercoralis* can live and develop in soil.		
4. The dry soil has a high risk of *S. stercoralis* infection.		
5. *S. stercoralis* can be detected in blood circulation.		
6. The symptom of strongyloidiasis consists of chronic diarrhea, flatulence, abdominal pain.		
7. *S. stercoralis* can distribute to lung, brain and spinal cord.		
8. The patient who use of steroids tends to develop hyperinfection syndrome and disseminated strongyloidiasis that are potentially fatal.		
9. Strongyloidiasis does not need treated because the illness will disappear.		
10. Although, the patients who infected with *S. stercoralis* were treated already. The patients can be infected with *S. stercoralis* again when them directly contact contaminated soil.		
11. The dog and cat are the reservoir hosts of *S. stercoralis* which can distribute parasite into environment.		
12. Strongyloidiasis can prevent by cooked meat consuming.		
13. The defecation into the cesspool is the prevention of *S. stercoralis* distribution.		
14. Taking stool as fertilizer does not cause to *S. stercoralis* distribution.		
15. Agriculturist is high risk to *S. stercoralis* infection.		

2.2 The attitude of *S. stercoralis* and strongyloidiasis prevention and control.

Topic	Attitude		
	Agree	Not sure	Disagree
**Prevention**			
1. Human who directly contacts contaminated soil has a chance to be strongyloidiasis.			
2. Wearing rubber gloves during farming can help prevent the infection of *S. setrcoralis*.			
3. Wearing boots can help prevent the infection of *S. setrcoralis*.			
4. After contact soil, the hand and /or feet washing can help prevent the infection of *S. Setrcoralis*.			
5. The stool examination is essential for strongyloidiasis diagnosis.			
6. Two or three times per year of stool examination is adequate for strongyloidiasis prevention and control.			
7. The patients who were found *S. stercoralis* were necessary to treatment.			
8. Currently, we have a good drug for *S. stercoralis* treatment. Therefore, the strongyloidiasis prevention and control was not necessary.			
9. We should take a drug for *S. stercoralis* treatment without stool examination.			
10. The strongyloidiasis patient who use of steroids tends to develop hyperinfection syndrome and disseminated strongyloidiasis that are potentially fatal.			
**Control**			
11. The defecation into the cesspool is the prevention of *S. stercoralis* distribution.			
12. Taking stool as fertilizer does cause to *S. stercoralis* distribution.			
13. Dog and cat are the reservoir hosts of *S. stercoralis*.			
14. Strongyloidiasis is not the public health problem. Therefore, this does not necessary to prevention and control.			
15. The stool examination in pets and pets' treatment is the strongyloidiasis prevention and control.			

Part 3: Risk behavior to *S. stercoralis* infection

**1. Have you ever defecated into surrounding environment?**	**Feces to envi**
( ) 1. NO	0 = no
( ) 2. Yes specific (area)..	1 = yes ………………..
**2. Have you ever directly contacted with contaminated soil?**	**Touching soil**
( ) 1. NO (pass no.3,4)	0 = no
( ) 2. Yes	1 = yes
**3. What was the activities with directly contacted contaminated soil without gloves?**	**Activity of touching soil**
....	…………………………
4**. How often you directly contacted contaminated soil without gloves?**	**Frequency of touching soil**
( ) 1. Everyday	1 = every day
( ) 2. 2-3 times/week	2 = 2-3 times/week
( ) 3. 1 time/week	3 = 1 time/week
( ) 4. 1 time/month	4 = 1 time/month
( ) 5. 2-3 times/month	5= 2-3 times/month
**5. Have you ever bare foot walking on contaminated soil?**	**Walking soil**
( ) 1. NO (pass no.6, 7)	0 = no
( ) 2. Yes	1 = yes.
**6. What were the activities with bare foot walking on contaminated soil?**	**Activity of walking soil**
....	……………………………
**7. How often your bare foot walking on contaminated soil?**	**Frequency of walking soil**
( ) 1. Everyday	1 = every day
( ) 2. 2-3 times/week	2 = 2-3 times/week
( ) 3. 1 time/week	3 = 1 time/week
( ) 4. 1 time/month	4 = 1 time/month
( ) 5. 2-3 times/month	5= 2-3 times/month

8. Where was area that you directly contacted or bare foot walking on contaminated soil

( ) 1. Own Residence

( ) 2. Own Farm

( ) 3. Others' farms

9. Have you ever use animal dung as fertilizer?

( ) 1 No (pass no.10)

( ) 2. Yes

10. How often you use animal dung as fertilizer?

( ) 1. 1 time/week ( ) 2. 2-3 time/week

( ) 3. 1 time/month ( ) 4. 2-3 months / time

( ) 5. 6 months / time

11. Have you ever use stool as fertilizer?

( ) 1 No (pass no.12)

( ) 2. Yes

12. How often you use stool as?

( ) 1. 1 time/week ( ) 2. 2-3 times/week

( ) 3. 1 time/month ( ) 4. 2-3 months / time

( ) 5. 6 months / time

13. Have you ever use steroid drug and herb at 3 month ago?

( ) 1 No (pass no.14)

( ) 2. Yes

14. How often you use steroid drug and herb at 3 month ago?

( ) 1 every day ( ) 2 2-3 times/week

( ) 3 1 time/week ( ) 4 1 time/month

( ) 5 2-3 times/month

Area of touching soil

1 = house area

2 = owner Farm or Fields

3 = other person’s Fields

<u>Use of animal fertilize</u>

0 = no 1= yes

<u>Use timing of animal fertilize</u>

1 = 2-3 times/week

2 = 1 time/week

3 = 1 time/month

4 = 2-3 times/month

5 = 6 times/month

<u>Use of human fertilize</u>

0 = no 1= yes

<u>Use timing of human fertilize</u>

1 = 2-3 times/week

2 = 1 time/week

3 = 1 time/month

4 = 2-3 times/month

5 = 6 times/month

<u>Use steroid</u>

0 = no 1 = yes

<u>Time of use steroid</u>

1 = every day

2 = 2-3 times/week

3 = 1 time/week

4 = 1 time/month

5= 2-3 times/month
