# Supplementary figures and images for: Impact of the health education and preventive equipment package (HEPEP) on prevention of Strongyloides stercoralis infection among rural communities in Northeast Thailand: a cluster randomized controlled trial
Source: BMC Public Health. 2018 Oct 19;18:1184. doi: 10.1186/s12889-018-6081-6 (PMC6194667; doi:10.1186/s12889-018-6081-6)

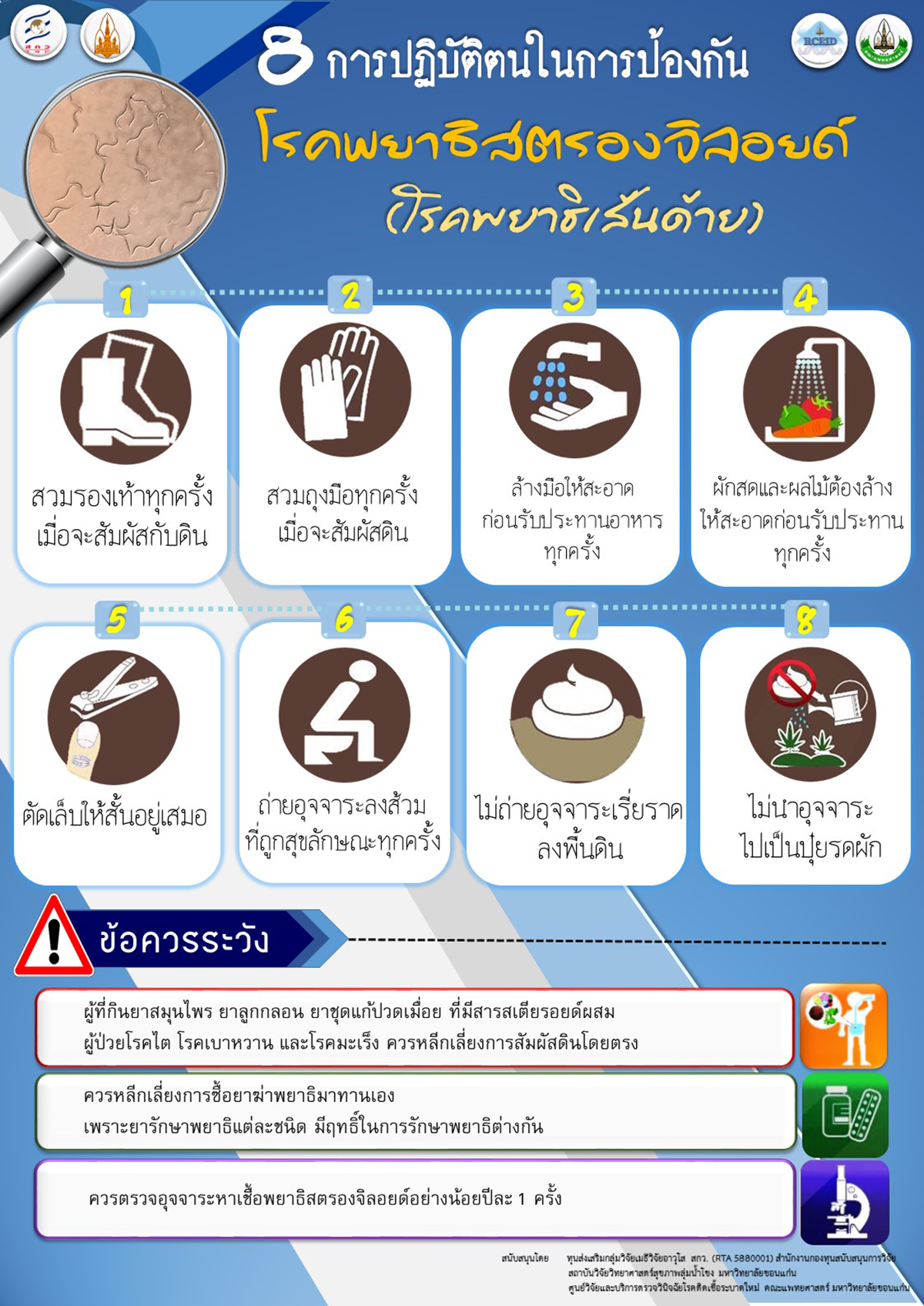

Supplement: Supplementary file 2 — Poster, a “Practices to Prevent Strongyloidiasis” poster. (TIF 1851 kb) [file 12889_2018_6081_MOESM2_ESM.tif]

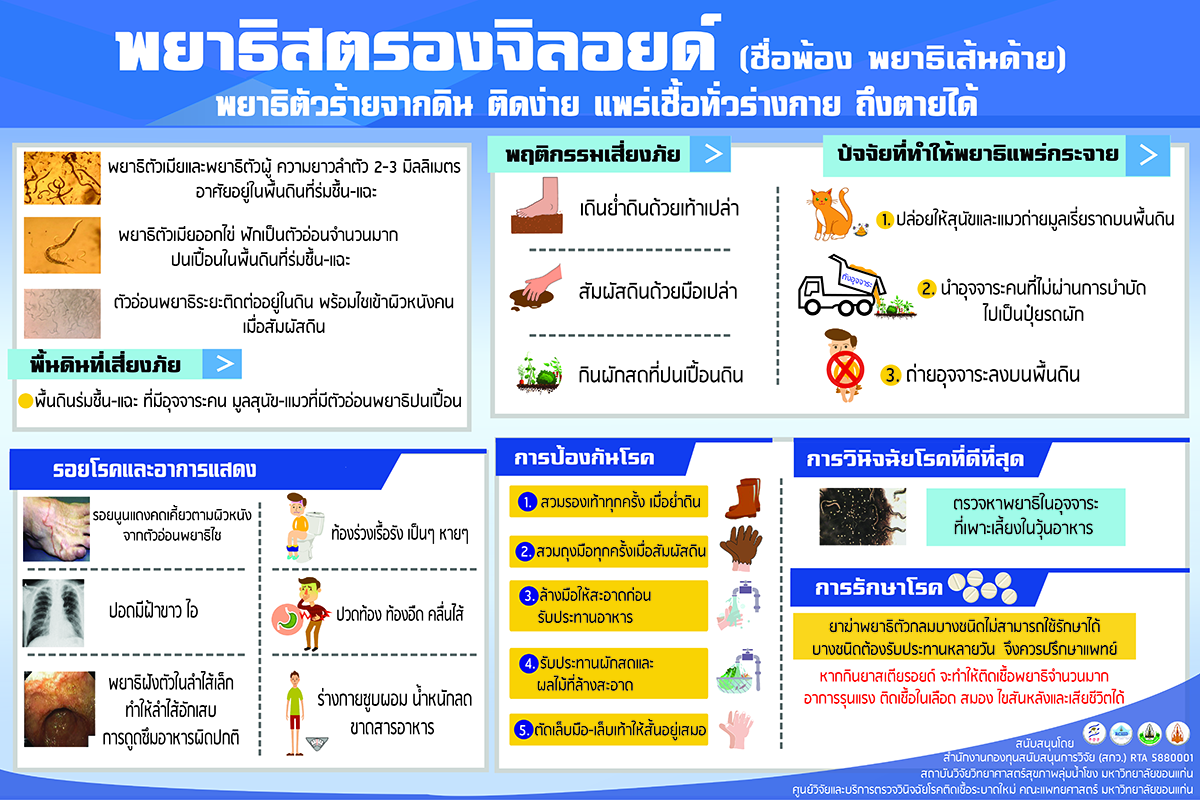

Supplement: Supplementary file 3 — Vinyl boards, a vinyl boards containing information on S. stercoralis and strongyloidiasis. (TIF 761 kb) [file 12889_2018_6081_MOESM3_ESM.tif]

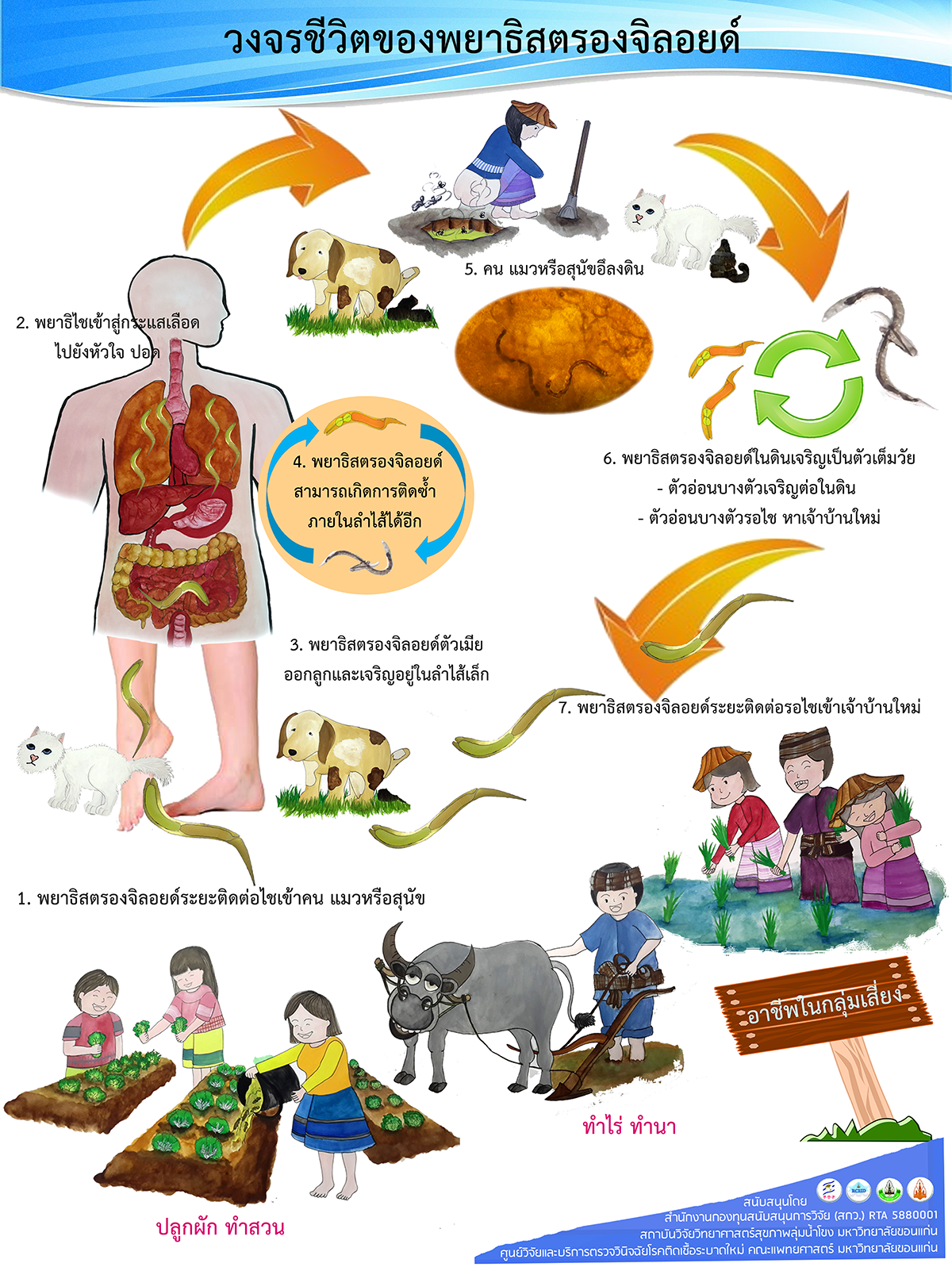

Supplement: Supplementary file 4 — Life Cycle poster, a “S. stercoralis Life Cycle” poster. (TIF 2012 kb) [file 12889_2018_6081_MOESM4_ESM.tif]

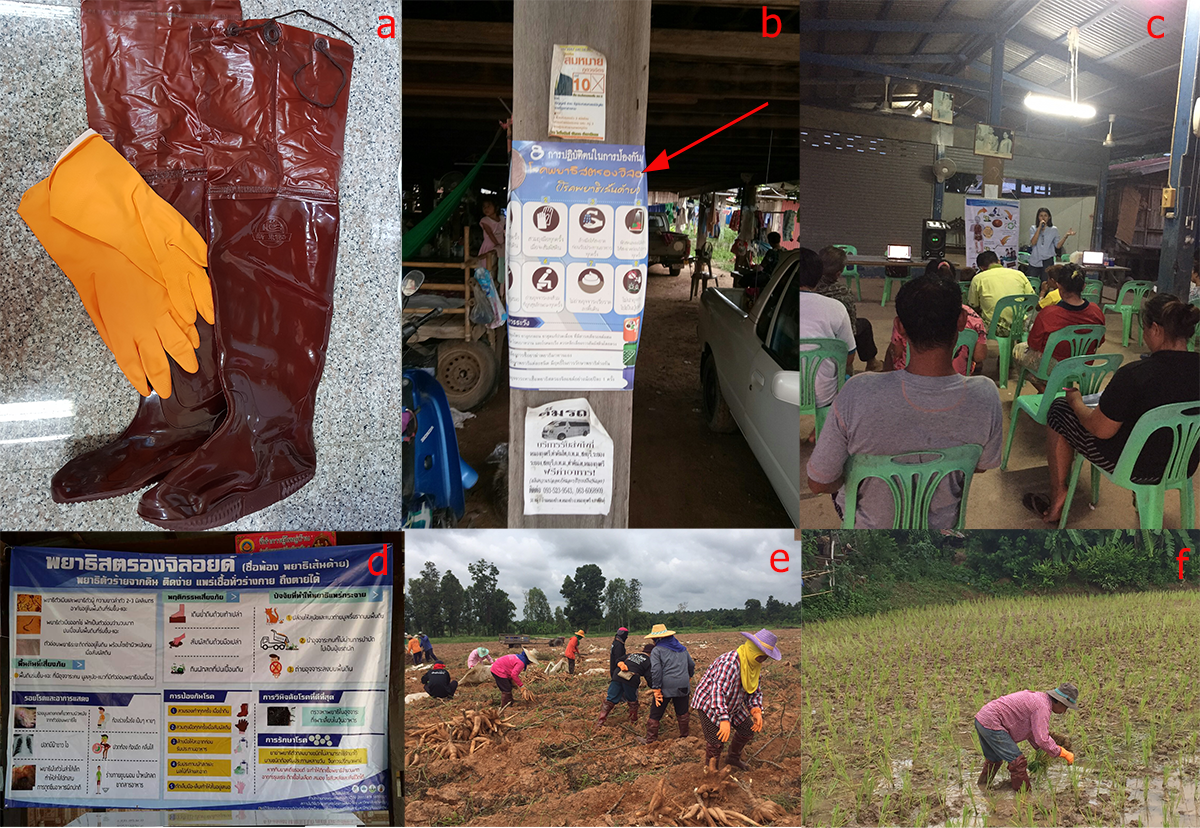

Supplement: Supplementary file 5 — The HEPEP-related follow-ups, a: equipment package (gloves and boots), b: “practice to prevent strongyloidiasis” poster, c: Lecture activity of human strongyloidiasis prevention by using S. stercoralis life cycle poster, d: S. stercoralis and strongyloidiasis advertising vinyl boards containing information on S. stercoralis and strongyloidiasis to promote in each village, e and f: checked equipment using every month by village health volunteers and researchers. (TIF 5464 kb) [file 12889_2018_6081_MOESM5_ESM.tif]
